# Using the R package *popharvest* to assess the sustainability of offtake in birds

**DOI:** 10.1002/ece3.11059

**Published:** 2024-04-01

**Authors:** Fred A. Johnson, Cyril Eraud, Charlotte Francesiaz, Guthrie S. Zimmerman, Mark D. Koneff

**Affiliations:** ^1^ Department of Ecoscience Aarhus University Aarhus C Denmark; ^2^ Office Français de la Biodiversité, Direction de la Recherche et de l'Appui Scientifique, Service Conservation et Gestion des Espèces à Enjeux Villiers‐en‐Bois France; ^3^ Office Français de la Biodiversité, Direction de la Recherche et de l'Appui Scientifique, Service Conservation et Gestion des Espèces Exploitées Juvignac France; ^4^ Division of Migratory Bird Management U.S. Fish and Wildlife Service Sacramento California USA; ^5^ Division of Migratory Bird Management U.S. Fish and Wildlife Service Orono Maine USA

**Keywords:** birds, density dependence, harvest, logistic model, management, objectives, offtake, *popharvest*, risk, sustainability, uncertainty

## Abstract

The R package *popharvest* was designed to help assess the sustainability of offtake in birds when only limited demographic information is available. In this article, we describe some basics of harvest theory and then discuss several considerations when using the different approaches in *popharvest* to assess whether observed harvests are unsustainable. Throughout, we emphasize the importance of distinguishing between the scientific and policy aspects of managing offtake. The principal product of *popharvest* is a sustainable harvest index (SHI), which can indicate whether the harvest is unsustainable but not the converse. SHI is estimated based on a simple, scalar model of logistic population growth, whose parameters may be estimated using limited knowledge of demography. Uncertainty in demography leads to a distribution of SHI values and it is the purview of the decision‐maker to determine what amounts to an acceptable risk when failing to reject the null hypothesis of sustainability. The attitude toward risk, in turn, will likely depend on the decision‐maker's objective(s) in managing offtake. The management objective as specified in *popharvest* is a social construct, informed by biology, but ultimately it is an expression of social values that usually vary among stakeholders. We therefore suggest that any standardization of criteria for management objectives in *popharvest* will necessarily be subjective and, thus, hard to defend in diverse decision‐making situations. Because of its ease of use, diverse functionalities, and a minimal requirement of demographic information, we expect the use of *popharvest* to become widespread. Nonetheless, we suggest that while *popharvest* provides a useful platform for rapid assessments of sustainability, it cannot substitute for sufficient expertise and experience in harvest theory and management.

## Graphical abstract

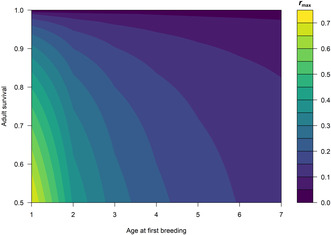

## 1 INTRODUCTION

Exploitation of birds by humans has a long history, with millions of birds taken worldwide for a variety of reasons, including for food, recreation, the pet trade, pest control, and as incidental take due to unrelated human activities (Shrubb, 2013). In many cases, if not most, the demography of exploited populations and the impacts of offtake are poorly understood. To address this challenge, the R package *popharvest* was designed to help assess the sustainability of offtake in birds when limited demographic information is available (Eraud et al., 2021). Because of its ease of use, diverse functionalities, and a minimal requirement of demographic information, we expect the use of *popharvest* to become widespread. In this article, we discuss what we believe to be important considerations when using *popharvest*, particularly for an audience who may not be well‐versed in harvest theory or management.

We emphasize that *popharvest* is simply a tool that makes methods developed by other authors more accessible. In particular, it builds on early work by Robinson and Redford (1991) on large mammals for estimating maximum rates of production based on age at first reproduction, fecundity, and maximum longevity. Slade et al. (1998) extended that work to incorporate empirical survival estimates. At about the same time, Wade (1998) introduced the potential biological removal (PBR) method to determine acceptable levels of incidental take of marine mammals:

(1) $$\mathrm{PBR}=N_{\min}\frac{R_{\max}}{2}F_{r}$$

where $N_{\min}$ is a minimum population estimate, $R_{\max}$ (equivalently, $r_{\max}$) is the maximum (i.e., intrinsic) rate of population growth, and $F_{R}$ is a recovery factor between 0.1 and 1. The term $R_{\max}/2$ is derived from the standard logistic model of population growth (i.e., assuming linear density dependence). It is the rate of offtake that maximizes the sustainable yield (MSY) while maintaining population size at half its carrying capacity. Thus, $F_{r}=1$ seeks to maintain a population at its level of maximum net productivity (K/2). Niel and Lebreton (2005) used a variation of PBR, defining the potential excess growth (PEG) as:

(2) $$\mathrm{PEG}=\mathrm{N\beta}\left(\lambda_{\max}-1\right)$$

where N is population size, $\left(\lambda_{\max}-1\right)=R_{\max}$, and β is a safety factor with 0.5 being a strict maximum. The PEG approach is implemented in *popharvest* with the safety factor β designated as $F_{s}$.

Runge et al. (2009) generalized the PBR approach to make it applicable to the full range of take scenarios and to better distinguish between scientific and policy elements of managing offtake. They called their approach potential take level (PTL):

(3) $${\mathrm{PTL}}_{t}=F_{O}\frac{r_{\max}}{2}N_{t}$$

where $0\leq F_{O}\leq 2$ is a factor that reflects management objectives; here $F_{O}=1$ represents the goal of MSY. Like PBR and PEG approaches, PTL is based on the standard logistic population model, but unlike the former approaches emphasizes that potential levels of take are dependent on population size $N_{t}$ that can change over time, *t*. All three approaches assume that carrying capacity and intrinsic growth rate are temporally constant. And, importantly, all three approaches assume that the population size is derived from a pre‐breeding survey or census and includes both breeders and nonbreeders. See Koneff et al. (2017) for a formulation of PTL that applies to post‐breeding populations.

An extended version of the PTL approach developed by Johnson et al. (2012) is available in *popharvest*. This approach accounts for various functional forms of density dependence:

(4) $${\mathrm{PTL}}_{t}=F_{O}\frac{r_{\max}\theta }{\left(\theta +1\right)}N_{t}$$

where θ>0 is the functional form of density dependence as either linear (θ=1), concave when viewed from below (θ>1), or convex (θ<1). It is this version of PTL that is available in *popharvest*, with $F_{O}$ represented as $F_{\mathrm{obj}}$.

The principal product of applications of *popharvest* is a sustainable harvest index (SHI), which is used to assess whether current harvest levels are unsustainable. SHI is calculated as the ratio of observed harvest to PEG or PTL, with values of SHI>1 indicating observed harvest is unsustainable relative to management objectives and/or risk tolerance. We emphasize, however, that the converse is not necessarily true. That is, values of SHI<1 are not conclusive of sustainability, analogous to a failure to reject the null hypothesis (i.e., harvest is sustainable). We are aware of only one published use of *popharvest*, in which Ellis and Cameron (2022) assessed the sustainability of waterbird harvests in the United Kingdom. However, there have been a number of applications that did not use *popharvest*, but did use PBR, PEG, or PTL approaches, including Koneff et al. (2017), Lormée et al. (2019), Runge and Sauer (2017), Watts et al. (2015), and Zimmerman et al. (2022).

In what follows, we first describe some basics of harvest theory, and then discuss several considerations when using the different approaches in *popharvest* to assess whether observed harvests are unsustainable. Generally, these considerations fall into one of three categories: (1) ecology, (2) management objectives, and (3) uncertainty and risk. Most of these considerations are discussed in the article describing the *popharvest* package (Eraud et al., 2021), and our goal here is to simply emphasize and elaborate on them. Our motivation for doing so was derived from several experiences we have had in assisting others to use *popharvest* (or its methods) and correctly interpreting their results.

## 2 INTRODUCTION TO HARVEST THEORY

The harvest of wildlife is predicated on the notion of reproductive surplus and ultimately on the theory of density‐dependent population growth (Hilborn et al., 1995). This theory predicts a negative relationship between the rate of population growth and population density (i.e., number of individuals per unit of limiting resource) due to intraspecific competition for resources. In a relatively stable environment, unharvested populations tend to settle around an equilibrium where births balance deaths. Healthy populations respond to harvest losses by increasing reproductive output or through decreases in natural mortality because more resources are available per individual. Population size eventually settles around a new equilibrium and the harvest, if not too heavy, can be sustained without threatening the breeding stock. Managers of recreational harvest often attempt to maximize the sustainable harvest by driving population density to a level that maximizes the reproductive surplus (Beddington & May, 1977).

These ideas can be expressed with the simplest of population models:

(5) $$N_{t+1}=N_{t}+N_{t}r\left(N_{t}\right)-N_{t}h$$

where $N_{t}$ is population size at time *t*, *h* is harvest rate, and $r\left(N_{t}\right)$ is a function describing how net reproduction decreases with increasing population size. Dividing through by $N_{t}$, we have:

(6) $$\frac{N_{t+1}}{N_{t}}=1+r\left(N_{t}\right)-h$$

For a harvest rate to be sustainable, we must have $N_{t+1}/N_{t}=1$, and after simplifying the equation, we arrive at a sustainable harvest rate of $h=r\left(N_{t}\right)$. This expresses the idea that a sustainable harvest rate (and therefore a sustainable harvest) is a function of population size. Thus, it is important to recognize that there is no unique harvest rate (or harvest) that is sustainable.

One of the most commonly used models to determine sustainable harvests for birds is the theta‐logistic model:

(7) $$N_{t+1}=N_{t}+N_{t}r_{\max}\left[1-{\left(\frac{N_{t}}{K}\right)}^{\theta }\right]-h_{t}N_{t}$$

where $r\left(N_{t}\right)$ has been replaced by $r_{\max}\left[1-{\left(\frac{N_{t}}{K}\right)}^{\theta }\right]$, K is carrying capacity (i.e., the maximum number of animals the environment can support), $h_{t}$ is a potentially time‐specific harvest rate, $r_{\max}$ is the maximum recruitment rate in the absence of density dependence, and θ is the functional form (i.e., linear or nonlinear) of density dependence. The theta‐logistic model lacks age structure (i.e., a so‐called scalar model) and so should be considered a first approximation if reproductive or survival rates are likely to be age‐specific. The harvest rate *h* and harvest *H* for maximum sustainable yield (MSY) are (Johnson et al., 2012):

(8) $$h_{\mathrm{MSY}}=r_{\max}\frac{\theta }{\left(\theta +1\right)}$$

(9) $$H_{\mathrm{MSY}}=r_{\max}K\frac{\theta }{{\left(\theta +1\right)}^{\left(\theta +1\right)/\theta }}$$

and the equilibrium population size *N* associated with MSY is:

(10) $$N_{\mathrm{MSY}}=K{\left(\theta +1\right)}^{-1/\theta }$$

For the standard logistic with linear density dependence (i.e., θ=1), the management parameters simplify to:

(11) $$h_{\mathrm{MSY}}=\frac{r_{\max}}{2}$$

(12) $$H_{\mathrm{MSY}}=\frac{r_{\max}K}{4}$$

(13) $$N_{\mathrm{MSY}}=\frac{K}{2}$$

**FIGURE 1:**
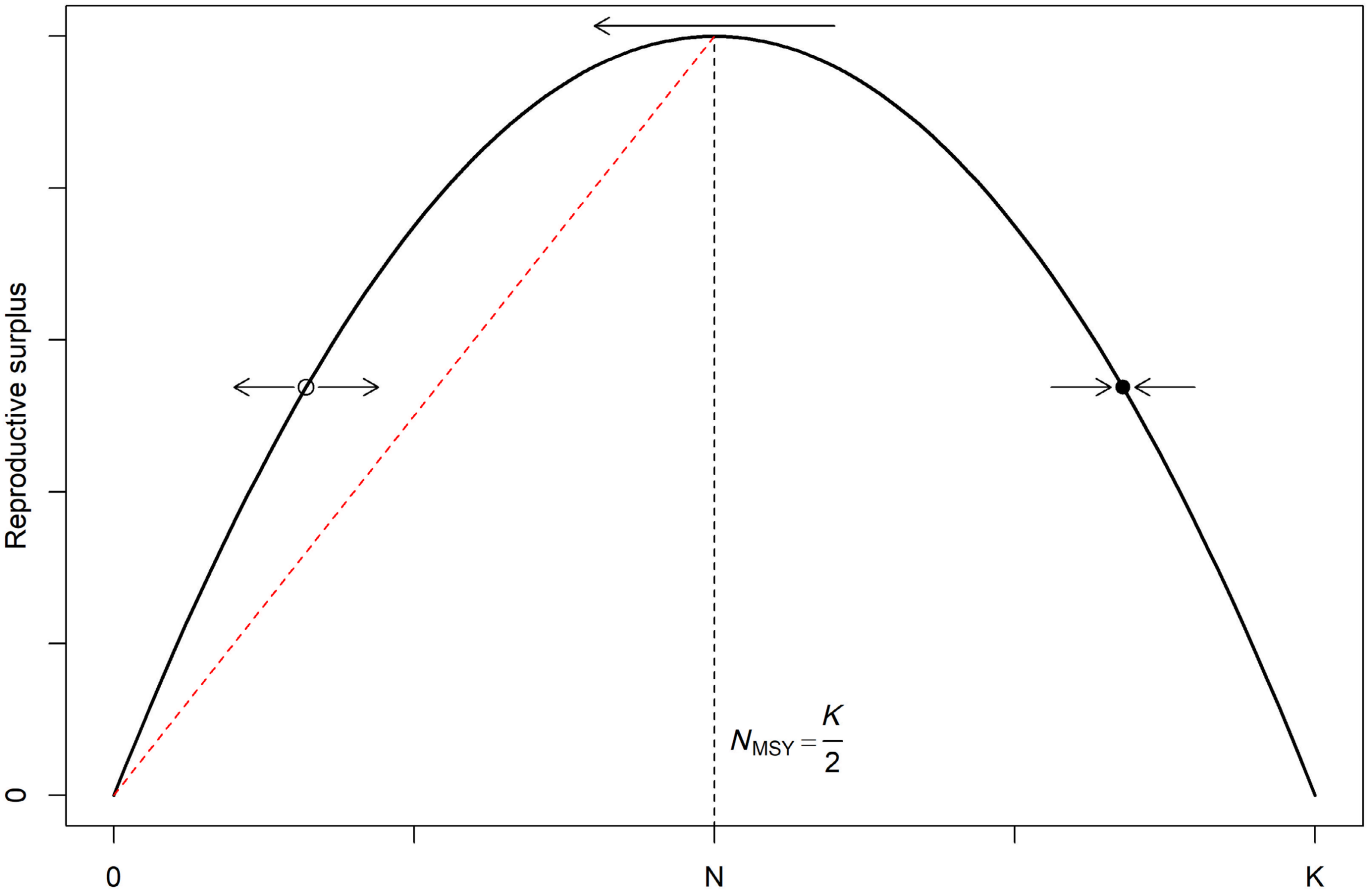
Reproductive surpluses as a function of population size, N, from the standard logistic model (i.e., linear density dependence). Equilibrium population sizes to the right of population size at maximum sustainable yield, $N_{\mathrm{MSY}}$, are stable (e.g., filled circle), while those to the left are unstable (e.g., open circle). *K* represents the unharvested population size (i.e., carrying capacity). The slope of the red dashed line is $h_{\mathrm{MSY}}=r_{\max}/2$.

Thus, in the standard logistic model, the maximum reproductive (i.e., harvestable) surplus is attained at a population level of one‐half carrying capacity. The sizes of the reproductive surpluses are parabolic with respect to population size (Figure 1). We note that equilibrium population sizes are stable for harvests below MSY; that is, harvests below MSY will always lead to an equilibrium population size greater than one‐half carrying capacity, irrespective of stochastic fluctuations in population size or harvest (Ludwig, 2001). However, equilibrium population sizes are unstable if population size falls below one‐half carrying capacity due to stochastic events and in that case even harvests < MSY can be unsustainable.

The scalar theta‐logistic model underlies computations of PTL in *popharvest* and setting $F_{\mathrm{obj}}=1$ implies MSY. In the PEG approach, if one is willing to assume linear density dependence, MSY is implied by setting $F_{s}=0.5.$

## 3 ECOLOGICAL CONSIDERATIONS

For both the PEG and PTL approaches, it is necessary to have an estimate of $\lambda_{\max}=\left(r_{\max}+1\right)$ or $r_{\max}$, the intrinsic finite and net rates of annual population growth, respectively. That is:

(14) $$N_{t+1}=N_{t}\lambda_{\max}\;\text{or}\;N_{t+1}=N_{t}+N_{t}r_{\max}$$

**FIGURE 2:**
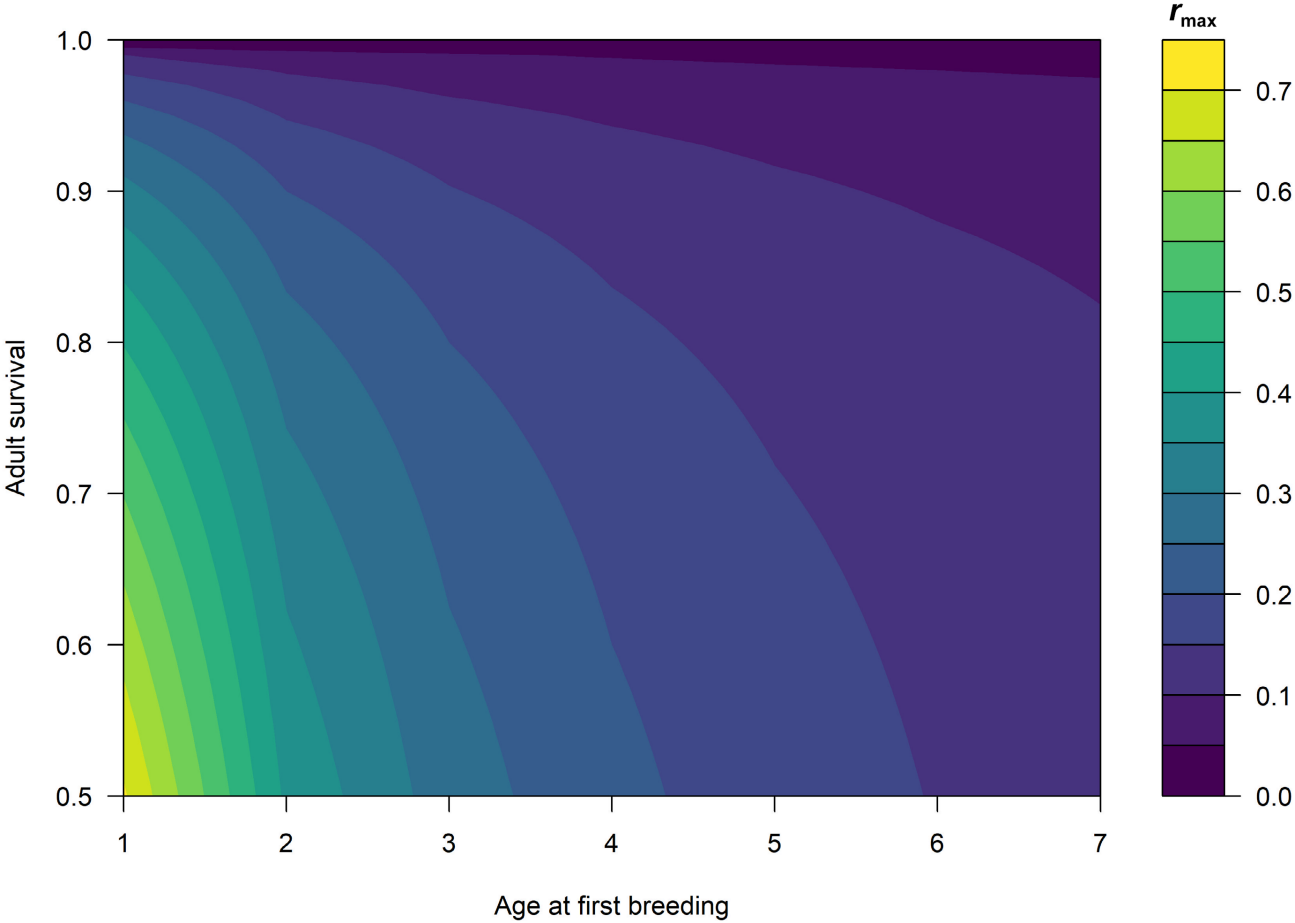
Sensitivity of the estimated intrinsic growth rate, $r_{\max}$, to adult survival and age at first breeding in birds based on Equation (17) from Niel and Lebreton (2005) (i.e., for “long‐lived” species).

These parameters will be unknown for most populations as they represent the rate of increase for populations under optimal conditions, absent any harvest or density‐dependent effects. An advantage of *popharvest* is that it allows these rates to be estimated using only knowledge of maximum adult survival and age at first breeding using the allometric relationships formulated by Niel and Lebreton (2005). The sensitivity of $r_{\max}$ to variation in maximum adult survival and age at first breeding is depicted in Figure 2. Generally, $r_{\max}$ is most sensitive to adult survival for birds that breed at an early age. For birds that first breed at greater than about 4 years, $r_{\max}$ is relatively insensitive to both adult survival and age at first breeding.

However, estimating the maximum (or intrinsic) adult survival may be as challenging as estimating $r_{\max}$. An approach available in *popharvest* is to use the method of Johnson et al. (2012), who demonstrated how intrinsic adult survival could be estimated using body mass and age at first breeding by relying on complete survival histories of birds in captivity (which was thought to mimic optimal conditions). When using this method, bird mass must be specified as a fixed value or a lognormal distribution in *popharvest*, for example by using the compendium by Dunning Jr. (2008). But a question arises as to whether one should use the mass of males or females because sexual dimorphism will induce different values of $r_{\max}$. Johnson et al. (2012) are silent on this question, but we suggest using both male and female body masses and calculating the mean mass as:

(15) $$\mu =\frac{\mu_{M}+\mu_{F}}{2}$$

and its variance as:

(16) $$\sigma^{2}=\frac{\sigma_{M}^{2}+\sigma_{F}^{2}}{2}+\frac{{\left(\mu_{M}-\mu \right)}^{2}+{\left(\mu_{F}-\mu \right)}^{2}}{2}$$

Although we have no empirical support for this recommendation, it may be better than arbitrarily picking a single sex for the analysis.

Users of *popharvest* should be mindful, however, that the allometric approaches for estimating $r_{\max}$ are derived in an evolutionary context and, thus, it is a maximum that may not be attainable under contemporary ecological conditions. Moreover, one cannot rule out the possibility that $r_{\max}$ or carrying capacity is changing over time due to large‐scale environmental forces such as climate change or ongoing conversion of landscapes. There is not likely anything one can do to account for this, other than to recognize that the use of $r_{\max}$ based on allometric relationships may overestimate a sustainable harvest level and therefore manage risk accordingly.

In using the allometric approach of Niel and Lebreton (2005), one must decide whether a species is “short‐lived” or “long‐lived,” and this can affect the magnitude of the estimate of $r_{\max}$. Unfortunately, Niel and Lebreton (2005) do not provide explicit guidance about how to make the distinction, although they only considered passerine species that breed at age 1 year as “short‐lived.” In any case, users of *popharvest* should be aware that the designation of a species as “short‐lived” will produce a higher value of $r_{\max}$ and, thus, suggest a higher level of sustainable harvest. For birds that breed at age one year, the difference in $r_{\max}$ from the “short‐lived” and “long‐lived” approaches can be substantial. For birds that breed at age two years, the difference in the two approaches yield differences in $r_{\max}$ <0.1. The differences in the two approaches for birds that breed at ≥3 years are generally negligible (<0.05). In keeping with Niel and Lebreton (2005), we suggest the “short‐lived” approach only be used for birds that breed at age 1 year.

We also note that survival estimates used to estimate $r_{\max}$ must be those attained under optimum ecological conditions (e.g., no density dependence and no harvest). Thus, empirical estimates of survival from the field may generate estimates of $r_{\max}$ (and sustainable harvests) that will be biased high. Finally, it is also important to recognize that the default procedure in *popharvest* is to assume the survival of juveniles is less than adults only for the first year of life. If that is not the case, the user must supply a mean value for juvenile survival for birds between age 1 year and breeding age (*α*), but here one must assume that survival is constant for all birds aged 1 to *α*−1 years.

**FIGURE 3:**
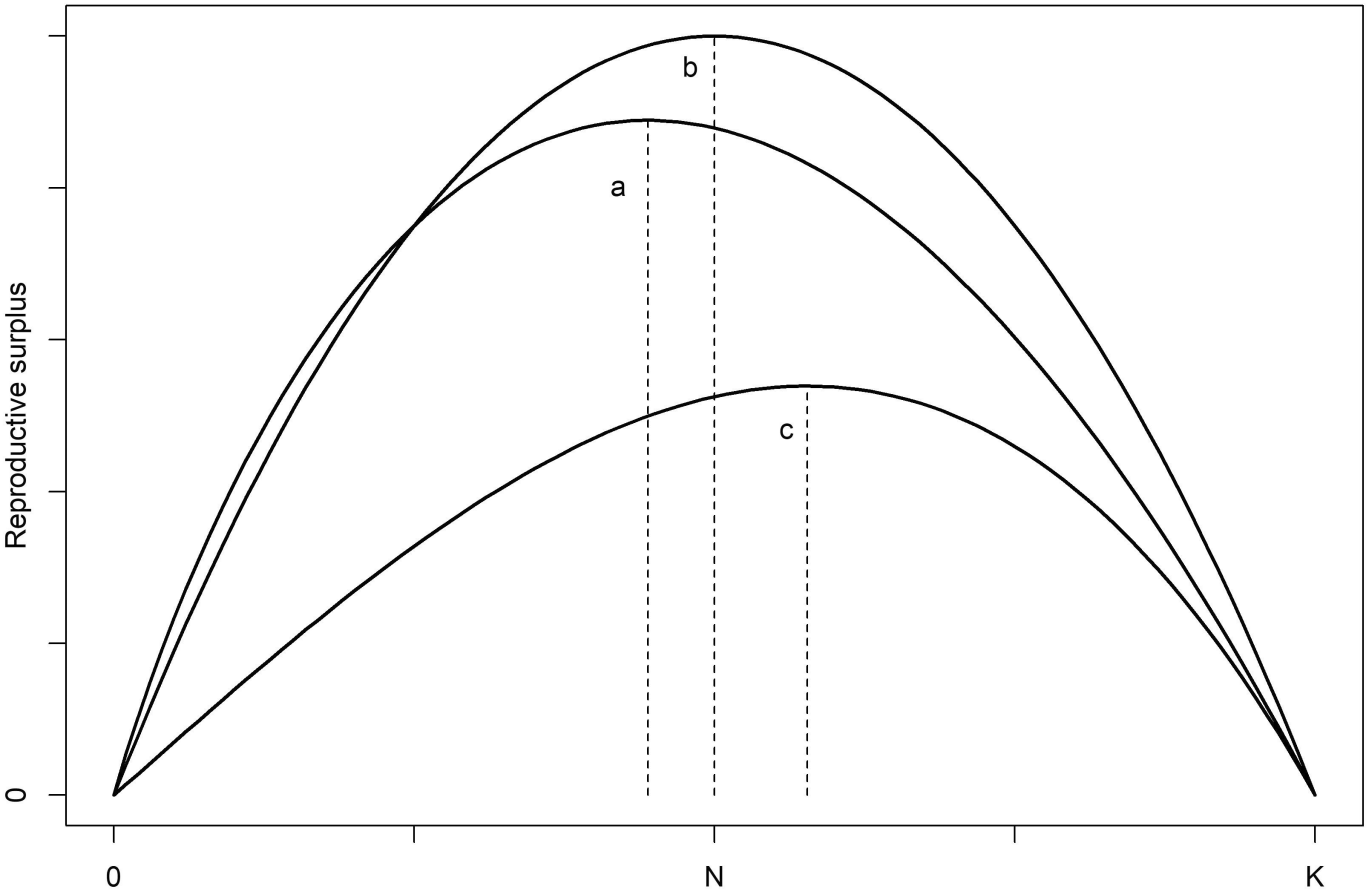
Reproductive surpluses in the theta‐logistic population model as a function of population size, *N*, when density dependence is (a) convex ($\theta =0.5,r_{\max}=1.5$), (b) linear ($\theta =1.0,r_{\max}=1.0$), or (c) concave $\theta =2,r_{\max}=0.35$. The vertical dashed lines indicate the equilibrium population sizes for maximizing sustainable harvests. *K* represents the unharvested population size (i.e., carrying capacity). The height of the curves (i.e., the size of the reproductive surplus) is controlled by $r_{\max}$ and the asymmetry of the curves by θ. In this figure, we recognize the inverse relationship between $r_{\max}$ and θ (Johnson et al., 2012).

The PTL approach assumes that density dependence operates to reduce the realized growth rate as population size approaches carrying capacity. The approach relies on logistic growth of a scalar population and posits that populations can “compensate” to some extent for harvest by increasing reproduction and/or decreasing natural mortality. The compensation effect as incorporated in the logistic model is phenomenological, in the sense that no specific survival or reproductive mechanisms are postulated (e.g., heterogeneity in survival, Cooch et al., 2014). The original PTL approach assumed linear density dependence (θ=1) (Runge et al., 2009), but Johnson et al. (2012) extended the approach to account for nonlinear density dependence. In these cases, θ>1 produces a concave population response (when viewed from below), where density dependence is strongest nearest carrying capacity. When θ<1, the population response is convex, where density dependence is strongest far away from carrying capacity. Users of *popharvest* should be aware that the functional form of density dependence (i.e., how growth rate declines as a function of increasing population size) can have a substantial effect on conclusions regarding sustainability (Figure 3). This can be problematic because the form of density dependence is typically the least understood and most difficult of all demographic parameters to estimate (Clark et al., 2010). In *popharvest*, one can choose to estimate θ based on its apparent relationship with $r_{\max}$ (Johnson et al., 2012), but application of the method adds a great deal of uncertainty to conclusions regarding sustainability. It may be wise to examine both linear and nonlinear forms of density dependence to determine the sensitivity of SHI (Koneff et al., 2017). We end the ecological discussion of PTL by noting that while it explicitly recognizes a form of “compensation” for exploitation, other forms of population response are overlooked. For example, it does not account for potential “depensation” (or the so‐called Allee effect; Stephens et al., 1999) where the population growth rate can be low even when populations are far below carrying capacity (but see Haider et al., 2017). The Allee effect is most likely to manifest itself in severely depleted populations.

There are several ecological considerations common to both PEG and PTL approaches. Both approaches rely on scalar models that do not account for any age structure in population demography or harvests. Significant age structure has important implications in terms of transient dynamics and population momentum (Koons et al., 2006). A failure to account for it can lead to spurious conclusions regarding the sustainability of harvest (Hauser et al., 2006; Niel & Lebreton, 2005). Significant age structure is typically associated with longer‐lived species. We note, however, that while geese are relatively long‐lived, there is at least one example demonstrating that scalar models may be adequate for assessing the consequences of harvest (Johnson et al., 2018).

Clearly defining a target population could help reduce the potential of unexpected consequences of applying PEG and PTL in local areas or for certain subpopulations. However, defining populations can be difficult due to coarse monitoring efforts or mixing of subpopulations when harvest occurs. Therefore, it is imperative that estimates of population size and harvest used to assess sustainability are both reliable and carefully aligned in time and space. This is especially critical in a European context because monitoring programs are extremely fragmented and sometimes produce biased estimates of population size or offtake (Aubry et al., 2020; Elmberg et al., 2006; Johnson & Koffijberg, 2021) and because flyways and populations are not always well‐defined (Davidson & Stroud, 2006). In North America, monitoring programs for game birds are quite advanced, but estimates of population size and offtake for nongame birds are tenuous at best. Therefore, we suggest caution is warranted in what appears to be an increasing use of PTL and *popharvest* for permitting the take of nongame birds in North America (Johnson et al., 2012; Runge & Sauer, 2017; Zimmerman et al., 2022). One must also be mindful that rapid assessments of sustainability are typically a “snapshot” in time and, thus, may not be reflective of sustainability over a longer period. Thus, we encourage users to estimate sustainable harvest for a range of population sizes. Finally, users of *popharvest* should be mindful that estimates of offtake should include crippling loss, and this is problematic because crippling rates are only rarely monitored (Clausen et al., 2017). For ducks in North America, harvest estimates are often inflated by 20% to account for unretrieved harvests (Johnson et al., 1993). Ellis et al. (2022) reported a crippling rate of 22% for ducks in Illinois, USA.

## 4 MANAGEMENT OBJECTIVES

Perhaps the most challenging application of the methods used in *popharvest* involves specification of the safety factor $F_{s}$ in PEG or the management objective $F_{\mathrm{obj}}$ in PTL. We cannot stress strongly enough that these F values are a social construct, informed by biology, but ultimately they are an expression of social values that usually vary among stakeholders. One of the difficulties users may have with the safety factor in PEG is that it confounds ecological understanding (e.g., presence of density dependence) and management objectives (e.g., risk tolerance) (Runge et al., 2009). Assessment of risk is the purview of decision‐makers and involves two components: (1) the probability of an undesirable outcome (e.g., unsustainable harvest) and (2) the perceived consequences (i.e., value) of that outcome. We may generally assume that the conservationists are averse to risk, but the degree of risk aversion is a choice for decision‐makers and is likely to be heavily context‐dependent. Dillingham and Fletcher (2008) suggest using criteria from the International Union for the Conservation of Nature and Natural Resources (IUCN) to set $F_{s}=0.5$ for “least concern” species, $F_{s}=0.3$ for “near threatened,” and $F_{s}=0.1$ for “threatened” species. However, these values are completely arbitrary and, more importantly, have not been sufficiently vetted among a large community of diverse decision‐makers. Moreover, categorization of species as, for example, “least concern,” also involves somewhat arbitrary criteria. The IUCN criteria may exclude some specific life history information which could lead to spurious conclusions regarding sustainability. We therefore suggest that any standardization of criteria for $F_{s}$ will necessarily be subjective and, thus, hard to defend in diverse decision‐making situations. Close coordination with the decision‐maker(s) is thus essential for defining appropriate F values.

The PTL approach provides a better distinction between ecological understanding and management objectives (i.e., between the scientific and policy aspects of managing offtake). Rather than ask “is harvest unsustainable?,” the PTL approach asks whether a given level of harvest is likely to meet management objectives for hunting opportunity and equilibrium population size. In the PTL approach, $0<F_{\mathrm{obj}}<\left(\theta +1\right)/\theta$ where $F_{\mathrm{obj}}=1$ represents a desire to attain the MSY. It is well‐known, however, that application of MSY in a variable environment is likely to be unsustainable (Ludwig, 2001). To extract only a specified proportion $p_{\mathrm{obj}}$ of the MSY, one can specify as an objective:

(17) $$p_{\mathrm{obj}}=\frac{H<\mathrm{MSY}}{\mathrm{MSY}}$$

and solve numerically for $F_{\mathrm{obj}}$ using:

(18) pobj=Fobj1+θ1−Fobj1θ

The associated equilibrium size of the harvested population as a portion of carrying capacity, K, is (Johnson et al., 2012):

(19) NK=1−Fobjθθ+11θ

As with $F_{s}$, we believe it would be difficult to standardize a protocol for specification of $F_{\mathrm{obj}}$ as it is the purview of the decision‐maker and will be context‐dependent. Specifying an acceptable F value for both the PEG and PTL approaches should always explicitly consider current and desired population sizes, intrinsic and observed population growth rates, the time required to meet management objectives, demographic uncertainty and risk tolerance, and possibly other considerations. Generally, however, $F_{\mathrm{obj}}=1$ might be considered for robust populations subject to recreational harvest, while $F_{\mathrm{obj}}<1$ might be appropriate for more vulnerable populations. Finally, $F_{\mathrm{obj}}>1$ might be appropriate for invasive populations or those causing significant socioeconomic conflicts.

## 5 UNCERTAINTY AND RISK

There are always uncertain demographic aspects in assessing harvest sustainability. Fortunately, *popharvest* provides tools to account for sources of uncertainty in estimates of intrinsic growth rate, population size, and harvest (e.g., Watts et al., 2015). We advise users of *popharvest* to take full advantage of these tools rather than specifying deterministic values, even if they are relatively well‐known. The admission of uncertainty in all aspects of applying *popharvest* will necessarily lead to relatively large uncertainty in the determination of sustainability, and any determination will likely be less conclusive than decision‐makers would prefer. However, explicit recognition of ecological uncertainty is essential to an honest and transparent appraisal of sustainability. Therefore, in confronting this uncertainty, the decision‐maker must take responsibility for explicitly stating their risk tolerance.

To use *popharvest* to determine whether offtake may be unsustainable, we can define risk as the probability that a particular level of harvest exceeds the SHI, where values of SHI>1 are to be avoided. But what makes for an unacceptable probability $P\left(\mathrm{SHI}>1\right)$? We can likely assume the decision‐maker will accept a lower probability (i.e., risk) if the population is small and/or declining rapidly. But, like other policy aspects of management decisions, an acceptable $P\left(\mathrm{SHI}>1\right)$ is the purview of the decision‐maker and will be context‐dependent.

One possible approach to standardizing the degree of risk acceptance is to rely on the concept of stochastic dominance (Canessa et al., 2016; Levy, 2016). The idea is that the decision‐maker should be able to describe their subjective attitude toward risk as being risk averse, risk neutral, or risk seeking. If we generally believe conservation decision‐makers will be risk averse, then the decision‐maker would like to avoid both a large variance and negative skewness in the distribution of possible outcomes. To apply this concept using the output of *popharvest*, one would have to postulate varying potential levels of harvest (including the observed harvest) and then compare the cumulative distribution functions of the stochastic outcomes of SHI for each. If, based on the concepts of stochastic dominance, the preferred choice of harvest is below that observed, a risk‐averse decision‐maker could conclude that the observed harvest is inconsistent with the management objective $F_{\mathrm{obj}}$ specified in the PTL (for a risk‐averse decision‐maker). Unfortunately, the ability to examine stochastic dominance does not exist in *popharvest* and would require ancillary programming. This feature may be included in subsequent updates of *popharvest*.

We offer a last brief comment about the fact that the PEG approach confounds ecological understanding and management objectives, or risk tolerance in this case. It has been suggested that the population size *N* used in the calculation of PEG should represent a minimum estimate to hedge against falsely concluding a harvest is sustainable (Wade, 1998). Thus, it potentially passes a decision about risk attitude to the ecologist responsible for estimating population size. Overall, we prefer the PTL approach to PEG, bearing in mind the need to carefully distinguish between scientific and policy aspects of decision‐making.

## 6 WORKED EXAMPLES OF POPHARVEST

We here provide examples of applying *popharvest* to three species of birds with varying life histories and management objectives. We also compare the *popharvest* results with those derived from more data‐intensive methods.

### 6.1 Black vulture (*Coragyps atratus*)

Black vultures cause significant socioeconomic damages in the eastern United States (Runge et al., 2009). To estimate a maximum allowable take to reduce population size and thus minimize damages, we used the PTL approach in *popharvest*. We set $F_{\mathrm{obj}}=1$, which would result in a population size of about one‐half of the carrying capacity. We used a mass of 2.159 kg (SD = 0.130) (Dunning Jr., 2008) and specified type.p and type.e as random effects in the survival estimating function (see Eraud et al., 2021 for details). We considered a range of ages at first breeding from 4 to 6 years (Runge et al., 2009). We allowed *popharvest* to estimate the form of density dependence, θ, based on its apparent relationship with $r_{\max}$. Finally, we specified a “long” living rate and used 20 thousand stochastic simulations of the PTL. In *popharvest* language, this translates into:
set. seed(1234)
PTL(pop. fixed=91190, Nsim=20000, NSp= 1, Fobj=1, mass.lognorm=TRUE, mean.mass=2.159, sd.mass=0.130, type.p = "random", type.e = "random", alpha.unif=TRUE, min.alpha=4, max.alpha=6, estim.theta ="random", living.rate="long", harvest. fixed=0)



The estimated median $r_{\max}$ was 0.12 (SD = 0.02) and median θ was 2.51 (SD = 4.92). Runge et al. (2009), who relied on more detailed demographic data for black vultures, reported an estimated value of $r_{\max}=0.11$, and they assumed the form of density dependence was θ=1. For a median population size of 91.19 thousand in Virginia (Runge et al., 2009), the median potential take level from *popharvest* was 7.46 thousand (SD = 2.24). The constant harvest rate for an objective of $F_{\mathrm{obj}}=1$ can be found using the detailed simulation results, *i*, from *popharvest* as:

(20) $$h_{\mathrm{MSY}}=\underset{i}{\text{median}}\left(r_{\max,i}\frac{\theta_{i}}{\left(\theta_{i}+1\right)}\right)$$

The median harvest rate for black vultures was $h_{\mathrm{MSY}}=0.08\;\left(\mathrm{SD}=0.02\right)$. We report median values because they are considered a better measure of central tendency than the means for skewed distributions (as will be the case for most parameter distributions in *popharvest*). The *popharvest* results suggest that 7000 could be used as a rough guide for an acceptable level of take for black vultures in Virginia, depending on the objectives and the risk attitude of the decision‐maker. Using a different initial population size (i.e., the lower bound of a 60% credible interval: 66,660), Runge et al. (2009) calculated allowable take at 3533.

### 6.2 Taiga bean geese (*Anser fabalis fabalis*)

Taiga bean geese in northern Europe provide important hunting opportunities, but the population suffered a decline in the late 20th century (Marjakangas et al., 2015). We again used the PTL approach. Using formulas (15) and (16), we calculated an average mass and SD (i.e., 3.0205 ± 0.339 kg) from mean values for males and females of 3.198 ± 0.302 kg and 2.843 ± 0.274, respectively (Dunning Jr., 2008). We also specified type.p and type.e as random effects in the function estimating survival from mass and specified age at first breeding as 2–3 years. We further specified the living rate as “long” and allowed *popharvest* to estimate the form of density dependence. Finally, we used estimates of population size of 40.96 thousand (SD = 3.33) and a harvest of 7.26 thousand (SD = 0.131) from 2000 based on an update of an integrated population model (IPM) by Johnson et al. (2020). We also examined estimates from 2020 after population size had increased to 62.16 thousand (SD = 2.02) and harvest had been reduced to 3.56 thousand (SD = 0.14). We again used 20 thousand stochastic simulations of the PTL. After getting estimates of θ from *popharvest*, we used Equation (18) to set $F_{\mathrm{obj}}=0.33$ to specify (for illustrative purposes) that only 50% of the MSY should be taken in light of the population decline. In *popharvest* language:
set.seed(1234)
PTL(full.option=TRUE, Nsim=20000, NSp= 1, Fobj=0.33, pop.lognorm=TRUE, mean.pop=40960, sd.pop=3330, mass.lognorm=TRUE, mean. mass=3.0205, sd.mass=0.339, type.p = "random", type.e = "random", alpha.unif=TRUE, min.alpha=2, max.alpha=3, estim.theta ="random", living. rate="long", harvest.lognorm=TRUE, mean.harvest=7260, sd.harvest=131)



The estimated median from *popharvest* for $r_{\max}$ was 0.18 (SD = 0.03) and for θ, it was 2.20 (SD = 4.30). For comparison, using the IPM developed by Johnson et al. (2020), the derived (within the IPM) estimate of median $r_{\max}$ was 0.25 (SD = 0.02) and for θ it was 3.48 (SD = 0.89). The median SHI from *popharvest* for the year 2000 population and harvest estimates was SHI=4.47 (SD = 2.74), which is strongly suggestive of unsustainability based on $F_{\mathrm{obj}}=0.33$. Moreover, there was a 100% probability that the observed harvest in 2000 was not in line with a management objective to take only half the MSY. By 2020, harvests of taiga been geese had been reduced by half, but harvest was still largely incompatible with an objective to take only half of the MSY (SHI=1.44;SD=0.87) with the probability of unsustainability at 91%. In contrast, if we had used in *popharvest* the values of $r_{\max}$ (lognormal) and θ (fixed) suggested by the integrated population model, then $\mathrm{SHI}=0.90\;\left(\mathrm{SD}=0.09\right)$ and the probability of unsustainability fell to 12%. We believe at least part of the discrepancy here is a result of not being able to specify in *popharvest* that the estimate of θ derived from the IPM should include sampling error. In any case, these results suggest a high degree of caution is warranted in drawing strong conclusions about the unsustainability of harvest based solely on the results of *popharvest*.

### 6.3 Rock ptarmigan (*Lagopus muta islandorum*)

The rock ptarmigan is the principal game bird in Iceland, and it has been the focus of extensive monitoring and research efforts. The Environment Agency of Iceland is currently updating a harvest management plan, which includes the development of IPMs and optimization of harvest strategies using stochastic dynamic programming (Marescot et al., 2013). We can compare the inferences from that work (F. A. Johnson, *unpublished data*) with those using *popharvest*. As before, we used a PTL approach. We again used formulas (15) and (16) to specify an average body mass and SD (i.e., 0.535 ± 0.47 kg) from the mass of each sex (male: 0.521 kg, SD = 0.038; female: 0.550 kg, SD = 0.050). We specified type.p and type.e as random effects in the survival‐estimating function. Ptarmigan are known to breed at age 1 year and so this value was fixed. We further specified the living rate as “short” and allowed *popharvest* to derive a stochastic estimate of the form of density dependence. We used the estimate of spring population size of 18.2 thousand (SD = 3.6) and harvest of 7.1 thousand (SD = 1.1) that were derived from the IPM during 2022 from the East hunting region. We set the management objective as MSY ($F_{\mathrm{obj}}=1$) and again used 20 thousand stochastic simulations of PTL. In *popharvest* language:
set.seed(1234)
PTL(full.option=TRUE, Nsim=20000, NSp= 1, Fobj=1, pop.lognorm=TRUE, mean.pop=18200, sd.pop=3600, mass.lognorm=TRUE, mean.mass=0.535, sd.mass=0.047, type.p = "random", type.e = "random", alpha. fixed=1, estim.theta ="random", living.rate="short", harvest.lognorm=TRUE, mean.harvest=7100, sd.harvest=1100)



The estimated median of $r_{\max}$ from *popharvest* was 0.62 (SD = 0.11) and the median θ was 0.99 (SD = 2.05), suggesting near linear density dependence. Using the same Equation (20) for $h_{\mathrm{MSY}}$ as for black vultures, the median $h_{\mathrm{MSY}}$ for ptarmigan was 0.30 (SD = 0.13) for a pre‐breeding population. Following Koneff et al. (2017), we can reformulate Equation (20) to assess $h_{\mathrm{MSY}}$ for a post‐breeding population, that is:

(21) $$h_{\mathrm{MSY}\;\text{post}-\text{breeding}}=\underset{i}{\text{median}}\left(F_{\mathrm{obj}}\times \left(r_{\max,i}\frac{\theta_{i}}{1+\theta_{i}\left(\theta_{i}+1\right)}\right)\right)$$

We then estimated a median $h_{\mathrm{MSY}\;\text{post}-\text{breeding}}=0.23\;\left(\mathrm{SD}=0.08\right)$. The median sustainable harvest index was $\mathrm{SHI}=1.33\;\left(\mathrm{SD}=1.47\right)$, suggesting the mean harvest of 7.1 thousand for a spring population of 18.2 thousand was potentially unsustainable. The probability that the SHI exceeded 1.0 was 72%. Using the integrated population model, we derived a much higher median $r_{\max}=1.42\;\left(\mathrm{SD}=0.60\right)$, which seems to arise because of the very high reproductive rate of rock ptarmigan observed in Iceland (the estimated adult survival was similar to *popharvest* and the integrated population model). For a post‐breeding population, optimization of a harvest strategy using the IPM suggested $h_{\mathrm{MSY}}=0.33\;\left(\mathrm{SD}=0.003\right)$. The realized post‐breeding harvest rates of ptarmigan from the IPM have averaged 0.24 (SD = 0.05) since 2005 (very similar to the post‐breeding harvest rate to maximize yield derived from *popharvest*) and the population has been stable or slightly increasing with a mean of 16.9 thousand (SD = 3.3), suggesting harvests have been sustainable. Here again, caution is warranted in drawing strong conclusions about the unsustainability of harvest based solely on the results of *popharvest*. This is especially true in light of the large uncertainties typical of both *popharvest* inputs and outputs, which in turn lead to relatively high probabilities that SHI indices exceeding 1.0.

As illustrated with these examples, we suggest that users of *popharvest* report all input parameters and selections of *popharvest* options (e.g., whether age at first breeding is fixed or stochastic). At a minimum, we suggest reporting the medians and standard deviations of key output parameters (e.g., $r_{\max}$ and θ), as well as the probability that SHI exceeds 1.0. As noted above, any honest assessments of uncertainty will necessarily suggest relatively high probabilities that SHI>1 because the distribution of SHI values will always be skewed to the right (i.e., values of SHI<0 are not possible). Finally, comparisons between the outputs of *popharvest* with more detailed demographic information or similar species should be made whenever possible to guard against precipitous conclusions.

## 7 CONCLUSIONS

We expect that the R package *popharvest* will encourage broader use of established methods for assessing the sustainability of offtake in birds, especially among conservationists and managers who may have limited expertise in harvest theory, decision analysis, and computer programming. However, its ease of use is also a disadvantage if the nuances of its application are not fully appreciated. In particular, we are concerned about the confounding of science and values that is all too common in conservation decision‐making (Pielke Jr., 2007). All conservation decisions involve both predicting and valuing outcomes. The first part is the “objective” role of scientists and the second part is the “subjective” role of society (or the decision‐maker as their representative). Thus, we urge caution in the use of the PEG method in which the distinction between these components is not as transparent as we believe it should be. The PTL approach, while better at separating ecological understanding and management objectives, nonetheless presents its own challenges in application. In particular, we believe it may be unrealistic to develop a standardized protocol for establishing $F_{\mathrm{obj}}$ values that are universally accepted within the ornithological community. An alternative for a rapid assessment of sustainability would be to set $F_{\mathrm{obj}}=1$ (i.e., MSY) and then flag those species with an unacceptably high $P\left(\mathrm{SHI}\geq 1\right)$ as warranting a fuller consideration of relevant social values among the decision‐makers responsible for regulating the offtake of that species.

The presence of uncertainty in demographic parameters, extant population sizes, and harvest should be fully acknowledged and reported in applications of *popharvest*. Where estimates of sampling variation are unavailable, the ecologist might seek expert judgment to help characterize the uncertainty. Helpful examples of this approach are provided by Koneff et al. (2017), Johnson et al. (2017), and Moore et al. (2022). Here, as in other aspects of stock assessments, the expert elicitation procedure should be completely transparent and follow acceptable protocols (Hemming et al., 2018; Morgan, 2014). Regardless of how it is specified, uncertainty in demography induces a distribution of SHI indices, which in turn characterize the risk of undesirable outcomes (i.e., a failure to meet management objectives). We may perhaps assume reliably that conservation decision‐makers are risk averse, but we should guard against risk aversion becoming an absolute expression of the precautionary principle, which elevates concern for a species status above all considerations. Indeed, if the precautionary principle were applied unthinkingly in harvest management, no level of harvest would be acceptable. Obviously, there is the need to carefully consider the risk attendant to a broader range of relevant social values (e.g., the potential for socioeconomic conflict) when assessing a decision‐maker's risk tolerance.

## AUTHOR CONTRIBUTIONS

**Fred A. Johnson:** Conceptualization (lead); investigation (lead); writing – original draft (lead); writing – review and editing (lead). **Cyril Eraud:** Conceptualization (equal); investigation (equal); writing – review and editing (equal). **Charlotte Francesiaz:** Conceptualization (equal); investigation (equal); writing – review and editing (equal). **Guthrie S. Zimmerman:** Conceptualization (equal); investigation (equal); writing – review and editing (equal). **Mark D. Koneff:** Conceptualization (equal); investigation (equal); writing – review and editing (equal).

## CONFLICT OF INTEREST STATEMENT

None declared.

## Data Availability

No data were used in production of this manuscript.
